# Effectiveness of COVID-19 vaccination in healthcare workers in Shiga Prefecture, Japan

**DOI:** 10.1038/s41598-022-22682-3

**Published:** 2022-10-21

**Authors:** Tokuhiro Chano, Tomoko Yamashita, Hirokazu Fujimura, Hiroko Kita, Toshiyuki Ikemoto, Shinji Kume, Shin-ya Morita, Tomoyuki Suzuki, Fumihiko Kakuno

**Affiliations:** 1 Department of Clinical Laboratory Medicine, Shiga University of Medical Science Hospital, Otsu, Shiga 520-2192 Japan; 2 Department of Nephrology, Shiga University of Medical Science Hospital, Otsu, Shiga 520-2192 Japan; 3 Department of Pharmacy, Shiga University of Medical Science Hospital, Otsu, Shiga 520-2192 Japan; 4 Shiga Prefecture Administration, Otsu, Shiga 520-8577 Japan

**Keywords:** Viral infection, Viral infection

## Abstract

This study, which included serological and cellular immunity tests, evaluated whether coronavirus disease 2019 (COVID-19) vaccination adequately protected healthcare workers (HCWs) from COVID-19. Serological investigations were conducted among 1600 HCWs (mean ± standard deviation, 7.4 ± 1.4 months after the last COVID-19 vaccination). Anti-SARS-CoV-2 antibodies N-Ig, Spike-Ig (Roche), N-IgG, Spike-IgM, and -IgG (Abbott), were evaluated using a questionnaire of health condition. 161 HCWs were analyzed for cellular immunity using T-SPOT^®^ SARS-CoV-2 kit before, and 52 HCWs were followed up until 138.3 ± 15.7 days after their third vaccination. Spike-IgG value was 954.4 ± 2282.6 AU/mL. Forty-nine of the 1600 HCWs (3.06%) had pre-existing SARS-CoV-2 infection. None of the infectious seropositive HCWs required hospitalization. T-SPOT value was 85.0 ± 84.2 SFU/10^6^ cells before the third vaccination, which increased to 219.4 ± 230.4 SFU/10^6^ cells immediately after, but attenuated later (to 111.1 ± 133.6 SFU/10^6^ cells). Poor counts (< 40 SFU/10^6^ cells) were present in 34.8% and 38.5% of HCWs before and after the third vaccination, respectively. Our findings provide insights into humoral and cellular immune responses to repeated COVID-19 vaccinations. COVID-19 vaccination was effective in protecting HCWs from serious illness during the original Wuhan-1, Alpha, Delta and also ongoing Omicron-predominance periods. However, repeated vaccinations using current vaccine versions may not induce sufficient cellular immunity in all HCWs.

## Introduction

During the coronavirus disease 2019 (COVID-19) pandemic, healthcare workers (HCWs) diligently dealt with patients with COVID-19 and restricted themselves from various aspects of their own daily lives to avoid transmitting the disease to the general population. Nevertheless, HCWs have been considered to be at an increased risk of the disease, owing to their occupational exposure to infected patients. In Japan, the seroprevalence of severe acute respiratory syndrome coronavirus 2 (SARS-CoV-2) among HCWs was reported to be relatively high^1,2^. Hence, HCWs and their relatives have often been considered carriers of the disease and discriminated against by the Japanese local communities due to fear of contracting the disease.

**Figure 1:**
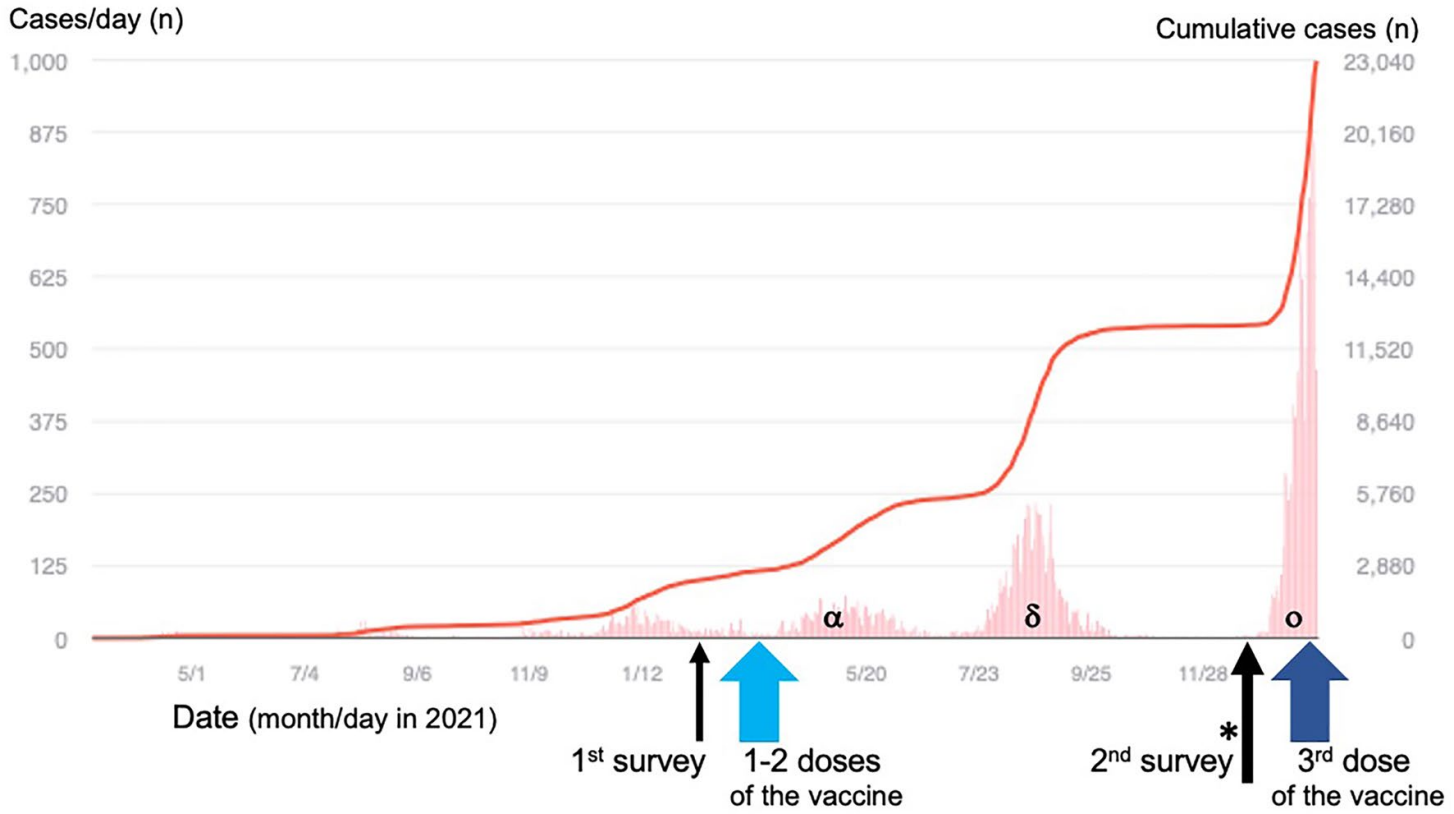
Numbers of new and cumulative cases of COVID-19 in Shiga Prefecture. New and cumulative cases of COVID-19 are indicated as bars and lines, respectively (https://covid2019.fa.xwire.jp/#japan_prefecture). The previous (1st) survey was performed in February 2021 (short black arrow), just before the initial COVID-19 vaccination of healthcare workers (wide light-blue arrow), and the present (2nd) survey was conducted in December 2021 (*, long black arrow), before the third dose of the vaccine (wide dark-blue arrow) and the ongoing sixth Omicron strain-predominant wave in Shiga Prefecture. Alpha, Delta and Omicron strain-predominant waves are indicated as α, δ and ο, respectively, in the graph. *Note*: COVID-19, coronavirus disease 2019; SARS-CoV-2, severe acute respiratory syndrome coronavirus 2.

To evaluate whether HCWs were at risk of COVID-19, the first serological survey for SARS-CoV-2 antibodies was conducted among 1237 HCWs in February 2021, approximately 11 months after the pandemic started in Shiga Prefecture when the seroprevalence among HCWs was not considerably higher than that of the general population^3^. Immediately after the first survey, to maintain healthcare infrastructures and protect healthcare personnel, the vaccination program against SARS-CoV-2 for HCWs was implemented from March 2021 to April 2021, ahead of the vaccination for the general population in Shiga Prefecture. Subsequently, the fourth and fifth waves of the disease caused predominantly by the SARS-CoV-2 Alpha and Delta strains, respectively (Fig. 1), emerged after the Summer Olympic Games (Tokyo 2020)^4^, forcing HCWs to continue with infectious disease control measures and routine medical care of patients under such adverse conditions.

In this study, a second serological investigation was conducted among 1600 HCWs in December 2021, in combination with a questionnaire survey on the health status of each participant to evaluate whether the vaccination rollout program adequately protected HCWs from SARS-CoV-2 in the absence of specific treatment for COVID-19. The first survey was conducted just before initiating the SARS-CoV-2 vaccination program for HCWs; thus, the participants' serological antibody levels were unaffected by the vaccine. The second (present) survey was conducted seven to eight months after the initial vaccination program, i.e., before the third dose of the vaccine and the ongoing sixth and seventh waves (Omicron strain-predominant ones) (Fig. 1).

As mentioned above, although the third booster of COVID-19 vaccines has been administered to maintain healthcare infrastructures and protect HCWs, it is still inconclusive as to whether these repeated vaccinations are effective over time^5–7^. This study aims to provide insight into humoral and cellular immune responses after repeated vaccinations over time in HCWs, by evaluating 161 participants for cellular immunity immediately before the third dose of the vaccine, rather than evaluating for specific antibody levels. Additionally, among these participants, 52 were followed up until maximally 175 days after the third vaccination.

## Methods

### Study design and participants

A cross-sectional study of HCWs was performed as a prefectural administrative investigation in 16 public hospitals designated for COVID-19 treatment in Shiga Prefecture, central Japan. The investigations were conducted between December 1, 2021, and December 28, 2021, which corresponded to a mean (± standard deviation) period of 7.4 (± 1.4) months after the administration of the last dose of an mRNA vaccine (BNT162b2 [Pfizer/BioNTech] or mRNA-1273 [Moderna]) and was approximately 10 months after the first survey conducted in February 2021^3^. We recruited 1,600 HCWs from 16 public hospitals. HCWs were invited to voluntarily participate in this study through advertisements and/or internal announcements. Those interested in the study were asked to contact the study team for an appointment.

### Data collection

Each consenting participant was provided a self-administered questionnaire (Supplementary File 1) to obtain their health conditions and history of exposure to SARS-CoV-2 seven to eight months after receiving the last dose of the mRNA vaccine. From each participant, 5 mL of peripheral venous blood was collected for the serological testing of SARS-CoV-2 antibodies, and 161 participants’ peripheral blood mononuclear cells (PBMCs) were collected from heparinized blood samples for evaluating cellular immunity.

### Serological tests for SARS-CoV-2 antibodies

The blood samples were separated by centrifugation (2500 × *g*, 10 min.), and the serum was frozen − 20 °C, less than 1 week until antibody evaluation. After all the study samples were collected, the serum samples were defrosted, and detection of SARS-CoV-2 antibodies was conducted using the Roche cobas^®^ 8000 (Roche, Basel, Switzerland) and the Abbott ARCHITECT^®^ i2000SR *PLUS* (Abbott, Chicago, IL) platforms at the Departments of Clinical Laboratory Medicine and Pharmacy, respectively, at the Shiga University of Medical Science Hospital. Roche cobas^®^ was used to measure serum antibodies specific to the SARS-CoV-2 nucleocapsid (Elecsys^®^ Anti-SARS-CoV-2 RUO; N-Ig) and the receptor-binding domain (RBD) of the spike protein (Elecsys^®^ Anti-SARS-CoV-2 S RUO; Spike-Ig)^8–12^. Abbott ARCHITECT^®^ assays were used to measure specific immunoglobulin (Ig) M to the spike protein (SARS-CoV-2 IgM; Spike-IgM), IgG to the nucleocapsid (SARS-CoV-2 IgG; N-IgG), and IgG to the RBD of the spike protein (SARS-CoV-2 IgG II Quant; Spike-IgG)^8,9,12,13^. The measured values of N-Ig, Spike-Ig, Spike-IgM, N-IgG, and Spike-IgG, adjusted with the manufacturers’ calibrators/standards, were interpreted as positive values, with a cut-off index of ≥ 1.0, ≥ 0.8 U/mL, ≥ 1.0, ≥ 1.4, and ≥ 50.0 AU/mL, respectively. Individuals who were serologically positive for antibodies specific to the SARS-CoV-2 nucleocapsid (N-Ig) were considered to have pre-existing SARS-CoV-2 infection.

### Evaluation of SARS-CoV-2T-cell responses in PBMCs

In addition to evaluating the values of Spike-IgG (Abbott) and Spike-Ig (Roche), cellular immunity was evaluated in 161 blood samples immediately before the third dose of the vaccine, and in 90 blood samples, after the third dose of the vaccine.

To measure SARS-CoV-2-specific T-cell reactivity, PBMCs were isolated from a heparinized whole blood sample using a Ficoll density gradient (Leucosep, #163288, Greiner, Germany). After quantification and dilution of recovered cells, 250,000 PBMCs were plated into each well of a T-SPOT^®^ Discovery SARS-CoV-2 kit (#DISCOVERY.432, Oxford Immunotec, Abingdon, UK). The kit is designed to measure responses to four different but overlapping peptide pools to cover protein sequences of four different SARS-CoV-2 antigens, including spike, nucleocapsid, and membrane proteins, without human leukocyte antigen restriction, and includes negative and positive controls. The fourth peptide pools showed a high degree of homology with endemic coronaviruses. The plated cells were incubated at 37 °C and 5% CO_2_ in a humidified chamber overnight, and we could detect circulating interferon-γ-secreting T cells, such as CD4 + helper T, CD8 + cytotoxic T and innate lymphocyte 1 cells. The sum of the T-SPOT immune response values was calculated (T-SPOT value), and > 48 and < 40 spot-forming units per 1,000,000 PBMCs (SFU/10^6^ cells) were reported as sufficient and poor counts, respectively^13,14^.

### Data analysis

To evaluate whether the questionnaire responses of each HCW were related to N-Ig seropositivity (infectious seropositivity) corresponding to pre-existing SARS-CoV-2 infection, binomial logistic regression analyses following univariate correlations were performed for each response. The questionnaire mainly included questions regarding health conditions before and after vaccinations (Supplementary File 1). Twenty-nine unvaccinated participants were excluded, and the association analysis with the infectious seropositivity was performed for 1571 participants who had been vaccinated at least once.

### Statistical analysis

The analyses were performed using Easy R software version 4.1.0^15^. Fisher’s exact test was used to assess the relationship between infectious seropositivity and questionnaire variables. Among variables with a *p*-value < 0.05 obtained using Fisher's exact test, binomial logistic regression was performed using the stepwise variable reduction method (Supplementary Data 1). For assessing the relation with T-SPOT values, multiple regression analysis was conducted using the stepwise variable reduction method with *p* < 0.05 as the level of statistical significance (Supplementary Data 2).

### Ethical statement

Written informed consent was obtained from all study participants. During the process of obtaining the consent, all participants were informed of the need to publish the results. All participants’ personal information was anonymized, and no participant is identifiable in the report. The Research Review Board of the Shiga University of Medical Science checked all the processes and contents of this study in accordance with relevant laws and regulations, and approved as an administrative investigation of Shiga Prefecture (No. R2021-039). The study findings have been disseminated to the participants (HCWs) through institutional representatives of the HCWs.

## Results

### Seroprevalence of antibodies against SARS-CoV-2 and participant characteristics

**Figure 2:**
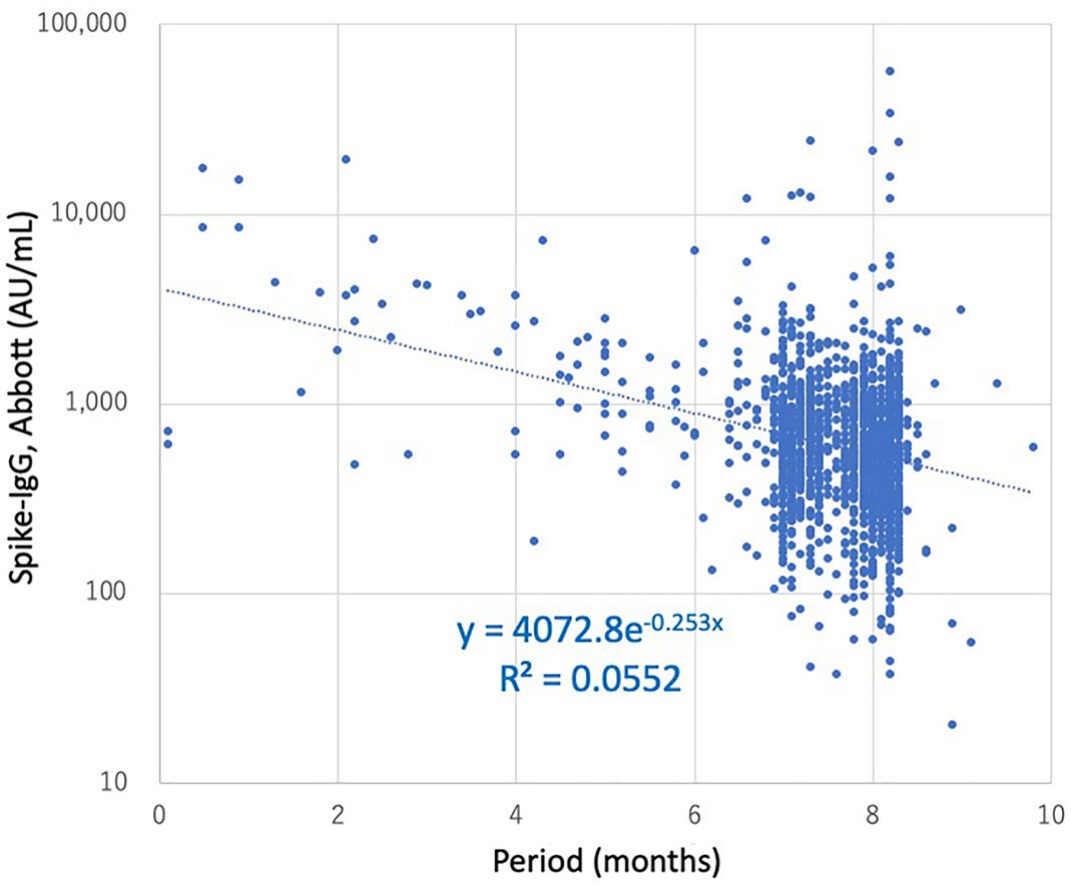
Values of Spike-IgG and periods since the last SARS-CoV-2 vaccination among healthcare workers. Values of Spike-IgG (IgG to the receptor-binding domain of the spike protein; SARS-CoV-2 IgG II Quant, Abbott) and periods since the administration of the last dose of the SARS-CoV-2 mRNA vaccine are indicated on the Y-axis (AU/mL) and on the X-axis (months), respectively (n = 1571). Data pertaining to the samples from 29 unvaccinated healthcare workers are not included in the graph. *Note*: SARS-CoV-2, severe acute respiratory syndrome coronavirus 2; IgG, immunoglobulin G.

This survey population of 1600 HCWs comprised 296 (18.5%) medical doctors, 984 (61.5%) nurses, 83 (5.2%) office workers, and 237 (14.8%) clinical laboratory technicians and physical therapists, etc. There was 66.2% female predominance (1059/1600) across the 16 hospitals. The cohort population was characterized by a mean age of 42.1 ± 16.4 years and a body mass index (BMI) of 23.4 ± 8.7 kg/m^2^. The number of participants who were unvaccinated, vaccinated once, vaccinated twice, and vaccinated thrice were 29, 6, 1561, and 4, respectively, and 1563 and 8 participants received BNT162b2 and mRNA-1273 vaccines, respectively. In this study, the time of blood collection was 7.41 ± 1.40 months from the administration of the last dose of the mRNA vaccine. The mean value of Spike-IgG (Abbott) in 1600 blood samples was 954.4 ± 2282.6 AU/mL (Fig. 2); however, the values of Spike-Ig (Roche) were saturated with > 250 U/mL in 1182 out of 1600 samples from this survey cohort, and this value was not precisely evaluated for all samples.

In the present study, Spike-IgG and -Ig didn't become indicators of the infectious seropositivity since the COVID-19 vaccination program had been implemented by the time of the second serological survey in December 2021. Eighteen participants who had a history of COVID-19 were infectious seropositive at the time of the first survey in February 2021. Among these 18 participants, 13 were negative for Abbott Spike-IgM, 16 were negative for Abbott N-IgG, and all were stably seropositive for Roche N-Ig, which is considered as serological reaction to reflect pre-existing SARS-CoV-2 infection that persisted until the second survey, and so the N-Ig positive individuals indicated specifically and persistently infectious seropositivity. Therefore, 49 out of 1600 participants (3.06%) were categorized as infectious seropositive. There were 31 (1.94%) new cases of SARS-CoV-2 infection between the first and second surveys. The questionnaire analysis revealed that among these 31 individuals, 8 had been clinically diagnosed with COVID-19, and 23 were considered asymptomatic and unaware that they were infected with SARS-CoV-2. There were no cases requiring hospitalization or cases with post-acute sequelae of COVID-19 among the 18 previously infected during the first survey, 8 newly diagnosed, and 23 asymptomatic but infectious seropositive cases.

### Risk factors associated with infectious seropositivity against SARS-CoV-2

Univariate analysis (Table 1) of SARS-CoV-2 infectious seropositivity (N-Ig) and individual characteristic variables of the self-administered questionnaire indicated statistically significant correlations among chronic respiratory diseases, smoking history, COVID-19 history, symptoms of common cold persisting for more than three days, and obese status (BMI > 30 kg/m^2^), in addition to a high level of each anti-SARS-CoV-2 antibody. Binomial logistic regression analysis of such statistically significant variables indicated that with a high level of each anti-SARS-CoV-2 antibody, only three factors, namely, chronic respiratory diseases (adjusted odds ratio [aOR], 1.93; 95% confidence interval [CI], 1.06–3.52; *p* = 0.0308), smoking history (aOR, 3.18; 95% CI, 1.53–6.63; *p* = 0.0020), and COVID-19 history (aOR, 22.7; 95% CI, 4.14–124; *p* = 0.0003; Table 1), were highly significant risk factors for infectious seropositivity.

**Table 1:** Participant characteristics and infectious seroprevalence of SARS-CoV-2.

Feature	Roche anti-SARS-CoV-2 nucleocapsid antibodies			Binomial logistic regression		
	Negative (n)	Positive (n)	*p*-value (Fisher’s exact test)	aOR	(95% CI)	*p*-value
**Chronic respiratory diseases: Q5**						
Applied: 2	7	1	0.0159	**1.93**	**(1.06–3.52)**	**0.0308**
In treatment: 3	13	2				
Untreated: 4	6	1				
Non-applied: 1	1496	45				
**Smoking history: Q15p**						
Applied: 2	343	19	0.0144	**3.18**	**(1.53–6.630)**	**0.0020**
Non-applied: 1	1179	30				
**History of COVID-19: Q19**						
Applied: 2	3	4	< 0.0001	**22.7**	**(4.14–124)**	**0.0003**
Non-hospitalized: 3	1	4				
Hospitalized: 4	0	0				
Non-applied: 1	1518	41				
**Common cold symptoms for more than three days: Q20**						
Non-applied: 1	1511	47	0.0032	0.250	(0.153–104.7)	0.4050
Applied: 2	10	0				
Non-hospitalized: 3	1	2				
Hospitalized: 4	0	0				
**Obese status: BMI (kg/m**^**2**^**)**						
BMI ≥ 30	241	14	0.0279	2.07	(0.914**–**4.69)	0.0809
BMI < 30	1281	35				
**Abbott anti-SARS-CoV-2 spike IgM**						
Positive: ≥ 1.0 AU/mL	47	15	< 0.0001	**8.48**	**(3.55–20.3)**	**< 0.0001**
Negative: < 1.0 AU/mL	1475	34				
**Abbott anti-SARS-CoV-2 nucleocapsid IgG**						
Positive: ≥ 1.4 AU/mL	32	12	< 0.0001	**9.78**	**(3.39–28.2)**	**< 0.0001**
Negative: < 1.4 AU/mL	1490	37				
**Abbott anti-SARS-CoV-2 spike-RBD IgG**						
High: ≥ 1000 AU/mL	320	41	< 0.0001	**17.5**	**(7.52–40.8)**	**< 0.0001**
Low: < 1000 AU/mL	1202	8				

*SARS-CoV-2* severe acute respiratory syndrome coronavirus 2, *aOR* adjusted odds ratio, *CI* confidence interval, *COVID-19* coronavirus disease 2019, *BMI* body mass index, *RBD* receptor-binding domain, *IgM* immunoglobulin M, *IgG* immunoglobulin G.

Binomial logistic regression that presents a significant difference is shown in bold.

### Cellular immunity before and after the third dose of the vaccine against SARS-CoV-2

The blood samples of the 161 HCWs evaluated using the T-SPOT cellular immunity test immediately before the third dose of the vaccine were collected 7.46 ± 0.88 months after the administration of the second dose of the vaccine. The mean value of Spike-IgG (Abbott) and of Spike-Ig (Roche) were 921.7 ± 845.4 AU/mL and 697.8 ± 591.5 U/mL, respectively.

**Figure 3:**
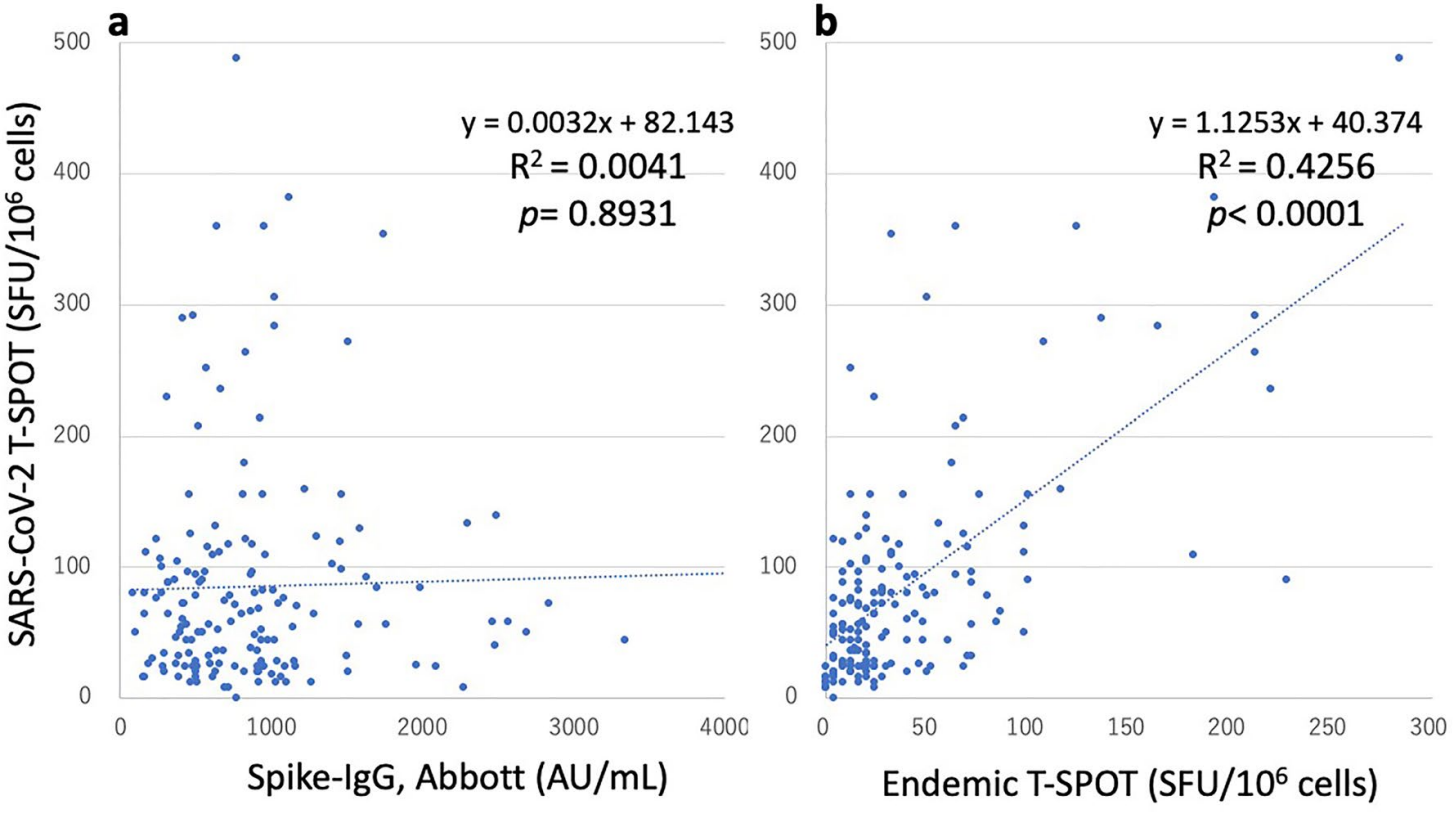
T-SPOT values for SARS-CoV-2 peptides did not correlate with the Spike-IgG values but correlated with the T-SPOT values for endemic coronaviruses. T-SPOT values for SARS-CoV-2 peptides (T-SPOT^®^ Discovery SARS-CoV-2 kit, Oxford Immunotec) are indicated on the Y-axis (SFU/10^6^ cells) in both panels (**a** and **b**). On the X-axis, the Spike-IgG values (AU/mL) and T-SPOT values for endemic coronaviruses (SFU/10^6^ cells) are indicated in a and b, respectively. *Note*: SARS-CoV-2, severe acute respiratory syndrome coronavirus 2; IgG, immunoglobulin G.

The T-SPOT value for SARS-CoV-2 peptides was 85.0 ± 84.2 SFU/10^6^ cells. However, 56 of the 161 (34.8%) samples had a poor count of < 40 SFU/10^6^ cells. The T-SPOT values did not correlate with Spike-IgG (Abbott) or Spike-Ig (Roche) against SARS-CoV-2 but correlated with the T-SPOT values for endemic coronaviruses (R^2^ = 0.4256, *p* < 0.0001; Fig. 3).

**Figure 4:**
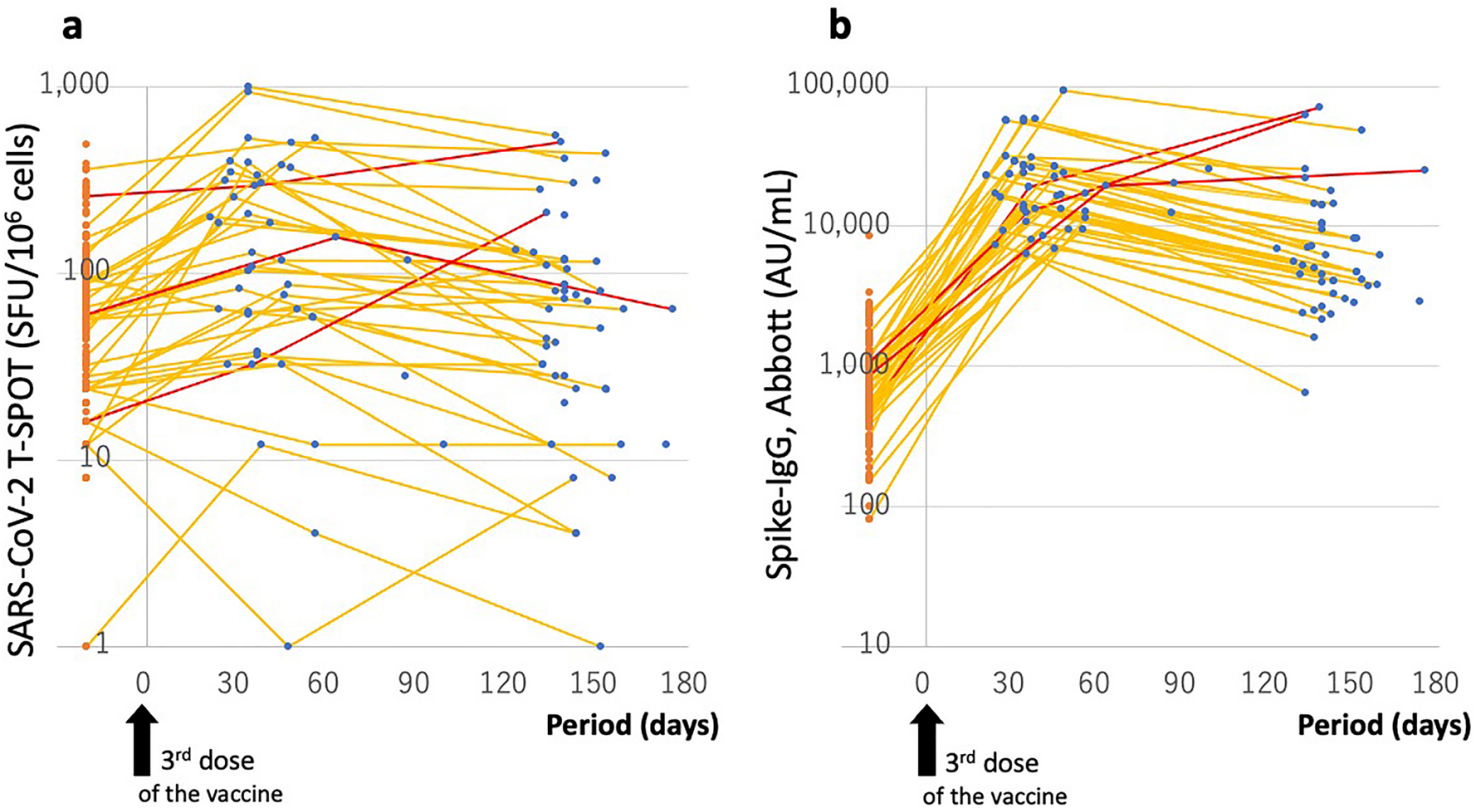
Time-elapsed values of T-SPOT for SARS-CoV-2 and those of Spike-IgG values before and after the third dose of the vaccine. Timings of the investigation before and after the third vaccination are indicated on the X-axis (days). On the Y-axis, the T-SPOT values for SARS-CoV-2 peptides (SFU/10^6^ cells) and Spike-IgG values (AU/mL) are indicated in (**a** and **b**), respectively. The 161 HCWs (orange dots) were evaluated immediately before receiving the third dose of the vaccine, and 52 of them were followed up for up to 175 days after receiving the third dose. Thirty-eight HCWs (yellow lines) were followed up twice after receiving the third dose, and a total of 90 blood samples (blue dots) were evaluated. Three breakthrough cases are indicated with red lines. The arrows indicate the day of receiving the third dose of the vaccine. *Note*: SARS-CoV-2, severe acute respiratory syndrome coronavirus 2; IgG, immunoglobulin G; HCWs, healthcare workers.

Additionally, the T-SPOT values of 52 of the 161 HCWs were followed up for up to 175 days after receiving the third dose of the vaccine. Thirty-eight HCWs had two follow-up tests at 38.7 ± 10.0 and 138.3 ± 15.7 days after the third dose, and a total of 90 blood samples were evaluated for cellular immunity. The participants experienced a mean increase of 258% in the T-SPOT value after the third dose from 85.0 ± 84.2 to 219.4 ± 230.4 SFU/10^6^ cells; however, the values later decreased to 111.1 ± 133.6 SFU/10^6^ cells. There was large variability in the individual T-SPOT values, and 20 of 52 HCWs (38.5%) had responses of < 40 SFU/10^6^ cells after the third dose of the vaccine, indicating poor cellular immunity (Fig. 4).

Spike-IgG (Abbott) values ranged from 921.7 ± 845.4 to 22,903.1 ± 17,820.8 AU/mL, indicting approximately 20- to 30-fold immediate increase induced by the third dose of the vaccine; however, these values later decreased to 11,494.4 ± 14,570.4 AU/mL (Fig. 4).

Among the 52 HCWs followed up after the third dose of the vaccine, three cases of breakthrough infection were identified. On detailed evaluation of their T cell responses, the three HCWs did not show additional enhancement of anti-spike T-SPOT values (32 → 36; 60 → 40; and 240 → 392 SFU/10^6^ cells) but experienced a substantial increase in their anti-nucleocapsid T-SPOT values (0 → 174; 0 → 24; and 8 → 108 SFU/10^6^ cells).

## Discussion

During the period between the first and second surveys (February 2021 to December 2021), in terms of the seroprevalence of SARS-CoV-2 among HCWs, the infectivity rate increased from 1.78 to 3.06%. During this period, the number of COVID-19 cases increased approximately five-fold, from 2467 to 12,477 in Shiga Prefecture^4^. The cumulative case number until December 28, 2021, was 12,477, which corresponded to 0.9% of the 1.41 million population in Shiga Prefecture, and the number of seropositive persons was presumably estimated as three to six times of that number. Thus, the antibody-positive rate in the general population should be 2.7–5.4%, which could not be factually assessed in this study. The infectious seropositivity rate (3.06%) among HCWs was also within this range and was not considered particularly high. Thus, this confirms that HCWs are at low risk of COVID-19 vaccine breakthrough infections^16^. In Shiga Prefecture, the risk of occupational infections caused by SARS-CoV-2 for HCWs was not higher than that for the general population; therefore, we conclude that HCWs should not be recognized by the local communities as having an increased risk for COVID-19.

Although 29 unvaccinated HCWs were included in this survey, none of them tested seropositive for infectious antibodies, and the vaccination rate among the HCWs was extremely high at 98.2%, suggesting that the unvaccinated HCWs were protected from SARS-CoV-2 infection due to a high level of herd immunity. HCWs are considered well-protected from SARS-CoV-2 infection with a higher vaccine coverage than that in the general population and intensive use of protective materials at work^16^. Among the 31 HCWs newly identified as infectious seropositive, 23 were considered asymptomatic and unaware of being infected with SARS-CoV-2. Including the 18 infected participants during the first survey, there were 49 infectious seropositive participants in this study; no participant with severe disease required hospitalization or experienced post-acute sequelae of COVID-19. Referring to the cumulative number of COVID-19 cases in Japan on April 18, 2022 (7,386,815; 473,672 requiring hospitalization and 29,041 deaths)^17^, it should be considered that one to four of the 49 infectious seropositive HCWs had severe disease requiring hospitalization, even if it depended on the SARS-CoV-2 strain, age of the patient, and presence of comorbidities. Based on the reduced number of SARS-CoV-2-infected HCWs in Shiga Prefecture, we believe that the vaccination rollout program for HCWs implemented between March 2021 and April 2021 was effective in protecting HCWs from symptomatic or especially severe illness, at least until the time of conducting the second survey, which was just before the ongoing Omicron strain-predominant waves. Meanwhile, it has been observed that vaccinated HCWs infected with SARS-CoV-2 usually present with minimal common cold symptoms. It is considerably difficult to detect such mild cases, and it may be inevitable that breakthrough clusters with SARS-CoV-2 infection occur in medical facilities via transmission of the virus from such patients, including infected medical personnel or the general public. Furthermore, HCWs may be at a greater risk of contracting COVID-19 from their colleagues or patients with undiagnosed early-stage COVID-19 than that arising from working in the infectious disease unit^18^. In the near future, it is desirable to implement control measures focusing on the prevention and treatment of severe cases, rather than those based on the number of COVID-19 cases, and in some cases, also to let infectious disease laws adapt to the social situations and different variants or strains.

In this survey cohort, Spike-IgG values decreased with a half-life of 50–60 days based on the period from the last vaccination (Fig. 2 and Supplementary Fig. 1), confirming the waning dynamics of humoral immunity to the SARS-CoV-2 vaccine over six months^19–22^. Smoking history was associated with infectious seropositivity, and the decrease in Spike-IgG value induced by the vaccination was persistently lower in the smoking group than that in the non-smoking group (Supplementary Fig. 2), which might have been associated with the smoking group’s susceptibility to the infection.

We have also noted some limitations to this study. This investigation included the predominant periods of the original Wuhan-1, Alpha, and Delta variants but not that caused by the Omicron variant; therefore, the results might not reflect the relevant characteristics of all SARS-CoV-2 strains. The sample size of this study might not be sufficient to evaluate the statistical correlations between multiple variables, and the design of this survey might not be sufficient to obtain conclusive causal evidence. Nevertheless, smoking habits and respiratory diseases are possible risk factors for SARS-CoV-2 infection.

It was suggested that cellular immunity to endemic coronaviruses might cross-react with SARS-CoV-2 and contribute to protection against hospitalization or death of COVID-19^5,23^. Cellular immunity induced after COVID-19 vaccination may remain stronger than humoral immunity^5^. This study were directly unable to measure neutralizing antibody titers or memory B cell numbers specific to the SARS-CoV-2 spike protein, but were able to measure the level of binding antibody, which correlates with the neutralizing antibody titers^24^. In fact, on our comparative evaluation of cellular and humoral immunities, humoral immunity had risen once but quickly decayed, while T-SPOT value, i.e., T-cell immune response, for SARS-CoV-2 peptides correlated strongly with that for endemic coronavirus and were more robust, although there were individual differences. HCWs who retained cellular immunity to endemic coronaviruses could effectively induced that to the SARS-CoV-2 spike protein by COVID-19 vaccination, and could likely maintain robust immunity.

During several months after the second or third dose of the vaccine, the binding antibody levels and neutralizing antibody titers are well proportional, and the former becoming a surrogate for the latter^24,25^. However, with prevalent strain like the Omicron variant, higher binding antibody levels are required to protect against the infection^24,26^. On the other hand, the cellular immunity induced by the current vaccines has been maintained even against the Omicron variant, and this is a likely contributing factor to not causing serious illness^27–29^. On analyzing humoral and cellular immunities before and after the third dose of the vaccine, we observed that repeated vaccinations using the present version of the vaccine for introducing the SARS-CoV-2 spike protein could only induce repeated activation and attenuation of humoral immunity, but could not strengthen cellular immunity or T cell memory in all HCWs, due to the large differences in the immune responses existing among HCWs (Fig. 4). Even for B cell memory, two doses of the vaccine can induce sufficient responses to various strains of SARS-CoV-2, and repeated vaccinations may not result in further reinforcement of long-term immunity^7,30^. In the present study, 3 HCWs with breakthrough infection after their third dose of the vaccine, who had highly Spike-IgG values (> 10,000 AU/mL; Fig. 4) that would have been sufficient to protect against infection with the original Wuhan-1, Alpha or Delta strain, could not prevent the Omicron strain infection, but presented only with minimal common cold symptoms, and did not cause serious illness required hospitalization. As suggested in previous papers^5,27–29,31^, cellular immunity elicited by two or three doses of the current COVID-19 vaccines has been maintained and cross-reacted also with the Omicron strain, so it seems that this strain infection did not cause severe diseases in these 3 breakthrough cases.

Meanwhile, these 3 breakthrough cases did not have evidence of an additional induction of T cell memory by a fourth repeated exposure to SARS-CoV-2 spike proteins; whereas, the breakthrough infection induced an immune response to the nucleocapsid protein. Thus, these results do not provide evidence that administering additional doses of the current COVID-19 vaccines would provide sufficient immune protection to the entire population, including HCWs, and vaccination alone will not be sufficient to end the COVID-19 pandemic. We suggest that other measures should be considered to complement the currently repeated vaccinations, such as introducing vaccines more adapted to the Omicron and future variants, which can be administered after three doses of BNT162b2^7^, or specific therapeutic agents against COVID-19 that can be freely prescribed by health care providers and covered by insurance.

In conclusion, this serological survey specific to SARS-CoV-2 infection among HCWs in Shiga Prefecture indicated that the seroprevalence among HCWs was not higher than that in the general population. This result suggests that healthcare facilities and workers are not at high risk for COVID-19 and should not be discriminated from local communities. The vaccination program was effective in protecting HCWs from serious illness during the original Wuhan-1, Alpha, Delta and also ongoing Omicron-predominance periods. However, COVID-19 breakthrough clusters may inevitably occur in various medical institutions in the near future. It is desirable to implement infection control measures placing more emphasis on the prevention and treatment of severe disease rather than on the number of infected patients. Repeated vaccinations using the current versions of vaccines may not induce sufficient immune protection in everyone; therefore, other measures that complement vaccinations are essential for ending the COVID-19 pandemic.

## Supplementary Information

Supplementary Information 1.

Supplementary Information 2.

Supplementary Information 3.

Supplementary Information 4.

## Data Availability

Supplementary data is available at www.nature.com/reprints. Consisting of data provided by the authors to benefit the reader, the posted materials are not copyedited and are the sole responsibility of the authors, therefore, questions or comments should be addressed to the corresponding author. The individual questionnaires used in this study have been submitted as Supplementary File 1. The datasets analyzed in the current study are available in Supplementary Data 1 and 2.
