# Supplementary material for: Effectiveness of COVID-19 vaccination in healthcare workers in Shiga Prefecture, Japan
Source: Sci Rep. 2022 Oct 21;12:17621. doi: 10.1038/s41598-022-22682-3 (PMC9586956; doi:10.1038/s41598-022-22682-3)
Supplement: Supplementary file 4 — Supplementary Information 4. [file 41598_2022_22682_MOESM4_ESM.docx]

**Table of Contents**

| Content | Page number |
| --- | --- |
| Supplementary Figure 1  (pdf) | **2** |
| Supplementary Figure 2  (pdf) | **3** |
| Supplementary File 1  (xlsx) | **4** |
| Supplementary Data 1  (xlsx) | **5** |
| Supplementary Data 2  (xlsx) | **6** |


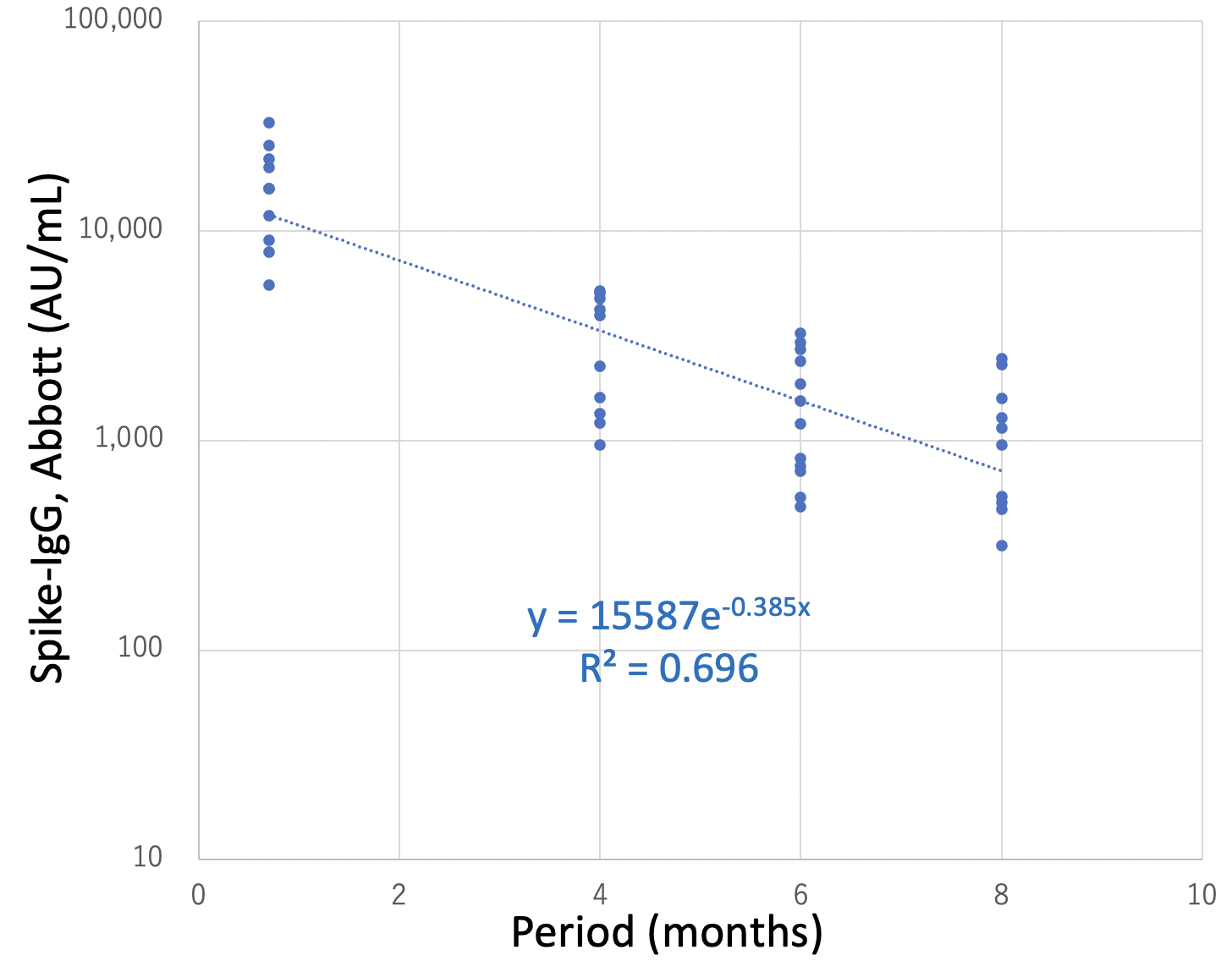


**Supplementary Figure 1.** Waning of titers of Spike-IgG over time since the initial two doses of SARS-CoV-2 mRNA vaccine.

Titers of Spike-IgG (IgG to the receptor-binding domain of spike protein; SARS-CoV-2 IgG II Quant, Abbott) decreased over time with a half-life of 50–60 days based on the period from the administration of the initial two doses of SARS-CoV-2 mRNA vaccine, BNT162b2, in 12 healthcare workers whose blood was routinely extracted. Values of Spike-IgG titers and periods are indicated on the y-axis (AU/mL) and on the x-axis (months), respectively. The blue dotted line is an exponential approximation line. *Note*. SARS-CoV-2, severe acute respiratory syndrome coronavirus 2

**
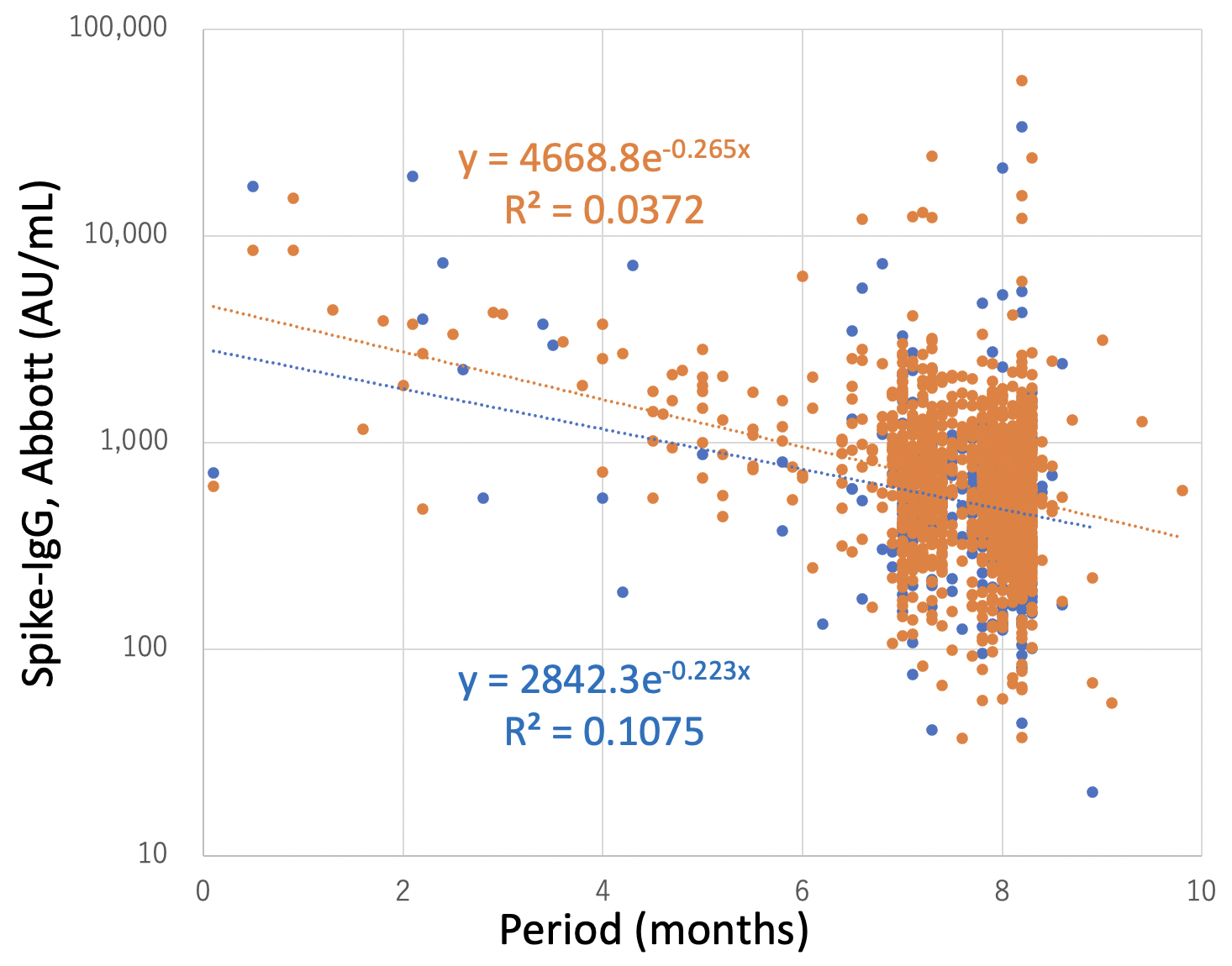
**

**Supplementary Figure 2.** Values of Spike-IgG titers and periods since the administration of the last dose of the SARS-CoV-2 vaccine among smokers and non-smokers.

Titers of Spike-IgG (IgG to the receptor-binding domain of spike protein; SARS-CoV-2 IgG II Quant, Abbott) and periods since the administration of the last dose of the SARS-CoV-2 mRNA vaccine are indicated on the y-axis (AU/mL) and on the x-axis (months), respectively (smokers, n = 366 [blue dots and dotted line]; non-smokers, n = 1,205 [orange dots and dotted line]). Data pertaining to the samples from 29 unvaccinated healthcare workers are not included in the graph. Blue and orange dotted lines are exponential approximation lines representing smokers and non-smokers, respectively. Note that the waning dynamics of Spike-IgG titers induced by the vaccination are likely to be lower in smokers than those in non-smokers. *Note*. SARS-CoV-2, severe acute respiratory syndrome coronavirus 2

**Supplementary File 1.** Individual questionnaire for assessing the health conditions of participants, before and after COVID-19 vaccination.

(xlsx)

*Note*. COVID-19, coronavirus disease 2019

**Supplementary Data 1.** Individual data regarding evaluated SARS-CoV-2 antibodies and health conditions of 1,571 healthcare workers in Shiga Prefecture.

(xlsx)

*Note*. SARS-CoV-2, severe acute respiratory syndrome coronavirus 2

**Supplementary Data 2.**  Individual data regarding T-SPOT values representing cellular immunity against SARS-CoV-2 and the antibodies and health conditions of 161 healthcare workers in Shiga Prefecture.

(xlsx)

*Note*. SARS-CoV-2, severe acute respiratory syndrome coronavirus 2
